# Digital sustainable transmission design strategies for regional culinary intangible cultural heritage based on cultural gene theory: a case study of Hubei Province, China

**DOI:** 10.3389/fnut.2026.1862554

**Published:** 2026-08-12

**Authors:** Wenting Cheng, Yeying Chen, Qiyun Deng, Ying Wang, Liwen He, Liang Huang

**Affiliations:** Sino-German Institute for Innovative Design, School of Industrial Design, Hubei University of Technology, Wuhan, Hubei, China

**Keywords:** culinary ICH, design strategy research, digitalization of ICH, regional culinary, sustainable transmission

## Abstract

**Introduction:**

The digital transmission of culinary intangible cultural heritage (ICH) faces several challenges, including difficulties in extracting cultural resources, organizing them into systematic categories, and overcoming monotonous forms of practical presentation. To address these issues, this study proposes a strategy for the extraction, organization, and design representation of culinary ICH resources. Grounded in cultural gene theory and integrated with living heritage theory, the strategy aims to support the sustainable transmission and design transformation of culinary ICH.

**Methods:**

The proposed strategy consists of three progressive stages. First, a five-level classification framework—“Region–City–Project–Category–Cultural Gene”—was developed to systematically deconstruct culinary ICH resources. Second, metadata annotation specifications were established to construct structured logical relationships among cultural data. Third, knowledge graphs, Sankey diagrams, and spatiotemporal evolution maps were integrated into a multidimensional visualization atlas to support knowledge-network interconnection, multi-project process comparison, and cultural transmission-path tracing. Culinary ICH resources from Hubei Province, China, were selected as a case study, and the proposed methodology was implemented through design practice and evaluated through a controlled experiment.

**Results:**

The evaluation results showed that the visualization atlas developed using the proposed strategy markedly improved target users’ ability to understand, retrieve, and compare culinary ICH resources. It also helped users identify the relationships among different cultural elements and gain a deeper understanding of their cultural meanings and contextual connections.

**Discussion:**

The findings demonstrate that integrating cultural gene theory with living heritage theory provides an effective framework for the classification, organization, and visual representation of regional culinary resources. The proposed strategy offers systematic support for the digital transmission and sustainable development of culinary ICH and provides a practical design reference for its digital presentation and creative transformation.

## 1 Introduction

Intangible cultural heritage refers to the forms of cultural heritage encompassing various cultural practices, representations, expressions, knowledge, and skills—as well as the instruments, objects, artifacts, and cultural spaces associated therewith (1). As a form of “living heritage” (2), the transmission and safeguarding of ICH can promote the sustainable development of culture in multiple dimensions. In China, centered on the goal of sustainable ICH transmission, exploring effective paths to support ICH safeguarding and transmission through digital means has become a crucial agenda for the continuity of the Chinese cultural lineage (3). Culinary ICH, as a cultural and socio-historical vehicle deeply embedded in daily practices, carries ethnic taste memories and life wisdom (4). The core of its sustainable transmission and safeguarding lies in its “living nature”—that is, allowing relevant information to achieve dynamic persistence through continuous social practice and research, rather than being archived as static specimens. The connotation of culinary ICH is reflected not only in physical attributes such as production techniques but also encompasses social relations, embodied knowledge, and long-accumulated cultural memories related to local traditional cooking methods (5) as well as the ongoing process of living transmission through constant practice, instruction, and re-creation. However, current digital technologies fail to accurately and systematically record the cultural connotations of culinary ICH, nor have they achieved corresponding design translation. This has led to risks such as the fragmentation of cultural contexts and the dissipation of technical details during the digital transmission of culinary ICH. Therefore, based on existing research on digital ICH transmission, it is necessary to explore digital sustainable transmission system design strategies that better align with the cultural characteristics of culinary ICH.

Digitalization of ICH refers to the process of collecting, recording, storing, modeling, and disseminating knowledge, skills, cultural information, and transmission processes related to ICH using digital technologies such as artificial intelligence, big data, and virtual reality (6). Its objective is to promote the sustainable utilization and trans-mission of ICH in digital forms. Digital technology enables the systematic archiving and in-depth research of ICH resources, helping heritage overcome spatial and temporal limitations in communication and interactive experiences. Consequently, it has gained increasing attention in recent years and has been applied to various ICH safe-guarding and transmission efforts. For instance, L. Cui utilized 3D scanning and photogrammetry to construct digital images, providing a high-fidelity digitalization path and display framework for the development and protection of Duanyun inkstones in Guangdong (7); Yidan Wang et al. employed artificial intelligence generated content technology to build a generative model for Lan Jia Xie, a traditional blue-and-white resist dyeing technique, and designed an interactive experience project for the Wenzhou Blue-and-White Resist Dyeing Museum (8); and Quan et al. addressed the fragmentation of regional ICH resources by proposing a cultural resource classification method based on knowledge graphs and deep learning that integrates semantic and visual in-formation (9). While explorations of these digital design methods and strategies help resolve difficulties in cultural resource extraction, categorical organization, and the monotony of presentation forms, most of these studies focus on static display-based ICH, such as patterns and artifacts. Research on digital sustainable transmission de-sign strategies specifically for culinary ICH remains relatively scarce. Static dis-play-based ICH follows a more intuitive perceptual logic with fixed digitalization standards and tangible physical references. In contrast, culinary ICH is characterized by complex cultural structures and diverse forms of expression, posing greater challenges for digital extraction, organization, and presentation. In particular, culinary ICH relies heavily on daily life practices and tacit knowledge, exhibiting a “living nature” in intergenerational transmission. Traditional digital workflows struggle to per-form deep deconstruction and structured expression of their complex cultural attributes. This leads to limitations in resource extraction, classification, information organization, and visualization design, thereby restricting the collection, entry, design transformation, and sustainable utilization of culinary ICH.

Digitalization of ICH refers to the process of collecting, recording, storing, modeling, and disseminating knowledge, skills, cultural information, and transmission processes related to ICH using digital technologies such as artificial intelligence, big data, and virtual reality (6). Its objective is to promote the sustainable utilization and trans-mission of ICH in digital forms. Digital technology enables the systematic archiving and in-depth research of ICH resources, helping heritage overcome spatial and temporal limitations in communication and interactive experiences. While these approaches help resolve challenges such as difficulties in cultural resource extraction and categorical organization, as well as the monotony of practical presentation forms, most of these studies focus on static display-based ICH, such as patterns and artifacts, research on digital sustainable transmission design strategies specifically for culinary ICH remains relatively scarce. Research on digital sustainable transmission de-sign strategies specifically for culinary ICH remains relatively scarce. Static dis-play-based ICH follows a more intuitive perceptual logic with fixed digitalization standards and tangible physical references. In contrast, culinary ICH is characterized by complex cultural structures, sensory experience modalities, and diverse forms of expression, posing greater challenges for digital extraction, organization, and presentation. In particular, culinary ICH relies heavily on daily life practices and tacit knowledge, exhibiting a “living nature” in intergenerational transmission. However, a comprehensive review of current digital culinary ICH practices reveals that their research objects are predominantly confined to a single category of traditional manual cooking techniques, with methodological strategies relying heavily on static literature database archiving or shallow visual displays (10). Traditional digital workflows struggle to per-form deep deconstruction and structured expression of their complex cultural attributes. This leads to limitations in resource extraction, classification, information organization, and visualization design, thereby restricting the collection, entry, design transformation, and sustainable utilization of culinary ICH.

To address the aforementioned issues, this research—from the perspective of sustainable cultural transmission—takes the regional culinary ICH of Hubei Province, China, as its research object. Aimed at culinary culture researchers, the study proposes a digital sustainable transmission design strategy for regional culinary ICH based on cultural gene theory and living heritage theory. This strategy integrates cultural re-source collection, resource classification, metadata standard formulation, metadata encoding and annotation, and cultural gene visualization maps. It aims to support the systematic extraction and classification of regional culinary ICH resources, as well as structured information organization and visualization design translation. This design strategy enables a more systematic and comprehensive capture of the cultural characteristics of culinary ICH, providing methodological and strategic support for its sustainable utilization.

## 2 Literature review

### 2.1 Research in the field of ICH digitalization

The digitalization of ICH facilitates the creation of digital archives, the construction of cultural resource databases, and the reconstruction of cultural practice scenarios, thereby promoting its presentation and application in the digital age. In recent years, diversified digital means have provided new technical implementation paths for the sustainable transmission of ICH. While enhancing the preservation precision of cultural heritage resource, these technologies have also broadened the avenues for its sustainable utilization and development (11).

The application of digital technology in the field of ICH primarily revolves around key stages such as data collection, recording, processing, storage, exhibition, and dissemination (7). This has led to the formation of extensive technical application work-flows and has achieved significant progress in enhancing the efficiency of collecting and processing ICH visual information, as well as expanding exhibition effects and communication reach. For instance, Pengpeng Cheng utilized machine learning methods such as Convolutional Neural Networks (CNN) to extract the cultural genes of paper-cutting art through image classification and pattern recognition, performing automated classification and storage to provide technical support for the systematic organization of visual ICH resources (12). Qianwen Yu et al. proposed a generative and application method for Miao embroidery patterns based on Stable Diffusion and Low-Rank Adaptation (LoRA) fine-tuning, successfully applying it to the mining of cultural characteristics in digital embroidery (13). Wu Yingyue et al. explored the technical application of Nanjing Yunjin brocade weaving techniques in ICH design through structural analysis and parametric design (14). Mingke Wang combined NFT digital twins with blockchain technology to explore the digital safeguarding, dissemination, and sustainable transmission paths of Miao silver jewelry craftsmanship with-in the context of the Metaverse (15).

However, most existing research on ICH digitalization targets heritage types with explicit visual characteristics and well-defined technical processes. For such heritage, the technical paths for image recognition, extraction, processing, and digital translation are relatively clear. In contrast, culinary ICH relies heavily on daily life practices, tacit knowledge, and living transmission. Digital technologies oriented solely toward visual materials collection struggle to systematically capture and organize culinary cultural resources, and they exhibit limitations in supporting the in-depth under-standing of cultural spiritual cores and innovative applications. Therefore, it is necessary to explore corresponding digital sustainable transmission systematic strategies tailored to the cultural logic and knowledge organization methods of culinary ICH.

### 2.2 Current research status of culinary ICH digitalization

Culinary ICH is a vital category of ICH. From the perspectives of cultural studies, such as sociology and anthropology, culinary ICH refers not only to the food itself but also encompasses the methods of cultivation, cooking, and consumption passed down through generations, which are deeply rooted in daily life and social interaction (5). The safeguarding of culinary ICH resources is regarded as an effective path toward achieving social and cultural sustainability goals (1, 16). It holds significant importance in the construction of regional cultural image and identity, the value of consumer experience, local sustainable development and social governance, as well as global cultural mobility and reconstruction (17–19). However, because culinary ICH relies heavily on bodily practices and tacit knowledge, exhibiting a profound dynamic and living nature in intergenerational transmission, traditional static archiving models struggle to completely capture its cultural core. Therefore, it is urgent to explore systematic digital dynamic preservation and sustainable transmission strategies tailored to culinary ICH to mitigate the risks of cultural context fragmentation and the dissipation of technique details during the digitalization process.

Currently, relevant academic research on culinary ICH transmission predominantly focuses on specific stages of digitalization. For instance, regarding the resource organization of culinary ICH, Wenyan Li et al. constructed a multimodal dataset of Chinese culinary culture containing both image and text information, providing a data foundation for the digital collection, encoding, and computational understanding of regional culinary cultural knowledge (20). Partarakis et al. applied semantic ontology modeling methods to build a platform system capable of representing the knowledge structure of Mediterranean culinary culture and supporting digital exhibitions (10). In terms of digital design translation, Huafeng Quan supported the cultural translation of Miao batik culture into the packaging design of Guizhou Red Sour Soup from the levels of cultural data analysis and symbol extraction by integrating natural language processing (NLP), Kansei engineering, and semiotics to construct a mapping model be-tween consumer perceptual needs and packaging design elements (21). Fan Wen utilized factor analysis to collect the ICH genes of Yexie Soft Cake and extracted a cultural symbol system of Traditional Chinese Medicine (TCM) wellness, achieving the innovative presentation of traditional cultural elements in the packaging design of Yexie Soft Cake (22). While these studies have explored issues such as resource collection, categorical organization, digital exhibition, and design translation of regional culinary ICH to a certain extent, they remain relatively fragmented overall. A systematic digital sustainable transmission design innovation strategy for culinary ICH has yet to be established. Consequently, their role in providing systematic guidance for the digital sustainable utilization of culinary ICH is limited, which constrains the innovative ap-plication and sustainable development of culinary ICH in the digital age.

### 2.3 Research on strategies for integrating cultural genes into the field of ICH digital design

Cultural genes are core cultural information units within a cultural system characterized by stability and identifiability (23), capable of continuous replication and dissemination through transmission and evolution (24). The conception of cultural genes was first proposed by Charles J. Lumsden and Edward O. Wilson in the 1950s (25). Subsequently, in the 1970s, British biologist Richard Dawkins formally introduced the concept of cultural genes, terming them “meme,” and systematically expounded upon the fundamental laws governing their transmission and evolution (24).

Cultural gene theory enables the deconstruction of complex cultural systems into more organized and hierarchical cultural information units. It has been widely applied in ICH digital design research, particularly during the stages of cultural resource ex-traction and classification. Early scholars, based on “meme” theory, pro-posed “cultural replication units,” treating explicit elements such as architectural forms and clothing patterns as basic hereditary units (26). Subsequently, the concept of implicit genes emerged, leading to a “dichotomy” of explicit and implicit genes to ex-pound upon the connotations of cultural genes. For instance, Hassan Alli et al. distinguished the explicit cultural genes of Zhuxian Town woodblock New Year prints—primarily consisting of patterns, colors, materials, and technical characteristics—from implicit cultural genes centered on historical and cultural symbolic meanings (27). Bu Jun et al. categorized the Southern Fujian “Chest-slapping Dance” into explicit genes including costumes, movement characteristics, and color language, and implicit cultural genes comprising cultural origins and connotations (28). However, relevant academic research based on this theory predominantly focuses on the structural extraction of basic cultural elements, overlooking the living characteristics inherent to culinary ICH. This oversight results in an inability to comprehensively capture its cultural connotations and transmission modes.

Living heritage theory emphasizes everyday life practices as the foundation and inheritors as the core (29), aiming to facilitate the dynamic transmission of ICH through continuous generation and coordination within specific cultural contexts. Capable of capturing the living characteristics of cultural heritage, living heritage theory is particularly suitable for research on the safeguarding and transmission of ICH characterized by practice and dynamism and has seen increasing application in recent years. For instance, Zheng Aocheng combined the connotations of living heritage theory with an analysis of the living evolution of sports ICH to propose digital living transmission strategies for sports heritage (30). Yang Yi analyzed the contemporary practices of Qian Opera inheritors in exploring typical paths for the living trans-mission of traditional drama (31). Valentina Vadi further pointed out that although food is ephemeral, its production, cooking, and sharing practices constitute a “living legacy” (32), which continues to shape the cultural memory of groups through inter-generational transmission. Introducing the concept of living heritage into research on the digital sustainable transmission of culinary ICH helps to better reveal the dynamic transmission mechanisms of culinary ICH within daily practices, thereby refining the systematic extraction, classification, and structured information organization of culinary ICH resources.

Based on living heritage theory, scholars have further proposed that cultural genes with living characteristics should be identified (33). From the perspective of living heritage, Wang Yi categorized the cultural genes of Gannan Tea-Picking Opera into morphological, behavioral, and contextual genes, proposing a living transmission strategy centered on gene activation (34); Liu Tuo emphasized the importance of in-heritors as the core subjects in the living transmission of ICH (35). These studies demonstrate that incorporating living genes into the processes of cultural gene extraction, classification, and encoding helps reveal the living transmission mechanisms of culture within practical contexts, addressing the insufficient attention paid to the dynamic transmission dimension by the traditional dichotomy of explicit cultural genes and implicit cultural genes. Regarding current classifications of culinary cultural re-sources, the most common methods only record production techniques and material information (36), or divide them into material, institutional, and spiritual levels (37), without involving classification methods for the living dimension. However, culinary ICH encompasses not only explicit operational information such as processes, raw materials, and tools but also contains a vast amount of implicit knowledge dependent on cultural connotations. Furthermore, it relies heavily on specific social practices and intergenerational instruction, being inseparable from the continuous participation and living practices of inheritors. As the knowledge carriers and inheritors of culinary ICH, the inheritors and their transmission modes intuitively reflect the living attributes of culinary culture within spatial and temporal flows. Therefore, it is necessary to incorporate living transmission genes centered on inheritors into the traditional extraction, classification, and encoding systems based on the dichotomy of explicit cultural genes and implicit cultural genes. This approach aims to more systematically and comprehensively capture the cultural characteristics of culinary ICH, addressing the deficiencies in current culinary ICH research regarding cultural resource classification dimensions, thereby providing a systematic resource foundation for its sustainable utilization.

## 3 Materials and methods

### 3.1 Methodological framework

To address the challenges in culinary ICH digital transmission—such as the difficulties in extracting and organizing cultural resources and the monotony of practical presentation forms—this research proposes a design strategy for the extraction, organization, and visual presentation of culinary ICH. Grounded in cultural gene theory and integrated with living heritage theory, the strategy specifically comprises three coherent stages: the “Five-Level Classification Method for Culinary ICH,” the metadata annotation specifications, and a multidimensional visualization map design that integrates knowledge graphs, Sankey diagrams, and spatiotemporal evolution maps.

**Figure 1:**
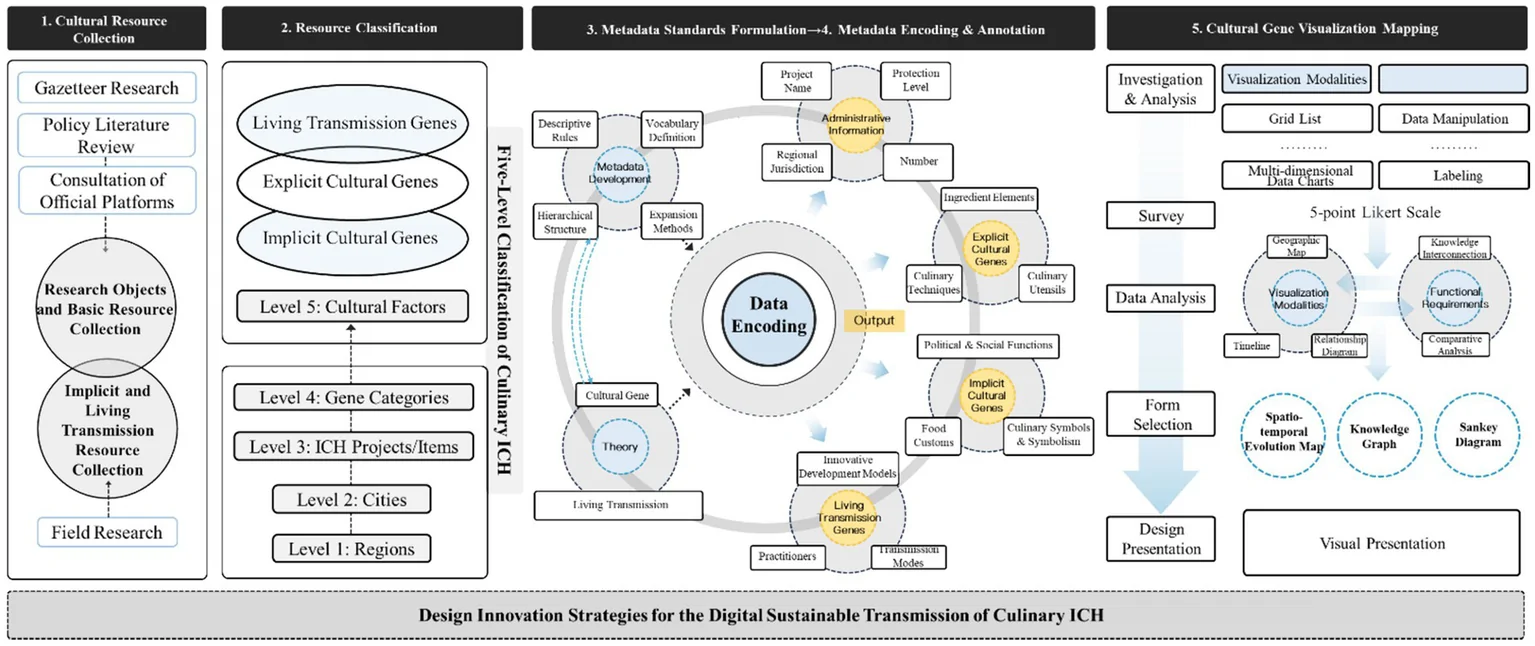
Design innovation strategies for the digital sustainable transmission of culinary ICH.

As shown in Figure 1, during the construction of the five-level classification method for culinary ICH, explicit cultural genes such as culinary techniques and implements; implicit cultural genes including food customs and symbolic meanings; and living transmission genes centered on inheritor information are integrated into a unified framework to systematically capture the cultural connotation of culinary ICH (see Table 1 for classification standards). In the metadata annotation stage, a five-level classification method and a structured in-formation organization strategy tailored to culinary ICH were formulated to achieve a systematic mapping of culinary cultural genes across the explicit, implicit, and living transmission dimensions. During the design presentation stage, this study referenced the data presentation formats under five categories of visualization techniques proposed by Daniel A. Keim (38) and the survey standards for cultural resource databases established by Liu Xiuru et al. (39). three specific visualization forms, namely knowledge graphs, Sankey diagrams, and spatiotemporal evolution maps, were selected based on questionnaire survey results, followed by the completion of their corresponding design presentations. Unlike previous ICH digital plat-forms primarily oriented toward public experience and cultural display, this strategy is targeted at professional users such as culinary culture researchers and designers, providing systematic resource support for the academic research and design reuse of culinary culture.

**Table 1:** Classification standards for culinary cultural genes.

Cultural gene category	Definition	Criteria for judgment	Characteristics	Concrete content
Explicit cultural genes	Genes that are clearly perceptible via sight, hearing, touch, etc., representing hereditary units with a high degree of phenotypic explicitness (23)	Culinary ICH information that can be obtained through observation, measurement, photography, or textual recording without relying on cultural interpretation	Concretization, high visibility, relative stability, ease of digital recording	Ingredient elements, culinary techniques, culinary utensils
Implicit cultural genes	Genes that are not easily perceptible via sight, hearing, touch, etc., representing hereditary units with a low degree of phenotypic explicitness (23)	Culinary cultural information that requires interpretation in combination with historical background, local culture, and social context	Strong abstraction, carrying symbolic meanings, relying heavily on cultural context	Food customs, political and social functions, culinary symbols and symbolism
Living transmission genes	Dynamic cultural genes that continuously persist by relying on the sustained practices of transmission subjects	Information that can only exist and continue through the sustained participation of inheritors, communities, or social activities	Dynamism, strong reliance on transmission subjects	Practitioners, transmission modes, innovation and development models

### 3.2 Research objects

This study focuses on culinary ICH projects at or above the provincial level in Hubei Province. Characterized by a dense network of rivers and numerous lakes, Hubei has long been known as a “land of fish and rice.” Under the long-term convergence and influence of Chu cultural traditions and the agricultural civilization of the Jianghan Plain, a diverse Jingchu culinary culture system with distinct regional characteristics has been formed (40). Culinary ICH in Hubei Province simultaneously encompasses a vast array of perceptible explicit cultural genes (such as ingredient elements, culinary utensils, and processing workflows), a rich and profoundly meaningful variety of implicit cultural genes (such as food customs, cultural symbols, and value systems), and living transmission genes that rely on the continuous practices of practitioners. It effectively presents the integrated correlation structure across explicit, implicit, and living transmission dimensions. Holding strong representativeness within the Chinese food culture system, it provides a solid sample foundation for exploring design strategies for the digital sustainable transmission of regional culinary ICH.

Although Hubei Province has established an ICH inventory and archiving system, its resource organization remains predominantly reliant on static modalities such as simple textual records, making it difficult to fully present the cultural correlations, technological logics, and transmission relationships among different projects. Meanwhile, systematic digital resource organization practices for Hubei’s culinary ICH are extremely scarce. Therefore, it is necessary to transform scattered culinary culture resources into a searchable, linkable, and visual online knowledge network through digital means, thereby enhancing the efficiency of preservation, research, and design transformation for Hubei’s culinary ICH resources.

Consequently, this study selects Wuhan Cai Lin Ji hot-dry noodles as a typical case for further methodological practice. As a unique local dish of Wuhan, Cai Lin Ji hot-dry noodle was included in the expansion list of the Representative Items of National Intangible Cultural Heritage in 2021 (41). Characterized by its speed, convenience, and high oil and carbohydrate content (42), the dish originated from the practical need to quickly replenish energy for manual laborers such as dock workers during a specific historical period. It serves as a concentrated reflection of Wuhan’s dock history and culture (43) and holds distinct regional cultural symbolic significance. With urban economic development and changes in social lifestyles, Cai Lin Ji hot-dry noodles have undergone a process of dynamic development, including flavor refinement and production workflow optimization. It has gradually transformed into a daily dietary choice for citizens, deeply integrating into the everyday lives of Wuhan residents, and continues to thrive through the daily practices of inheritors and employees (43). Wuhan Cai Lin Ji hot-dry noodle exemplifies the dynamic evolution of culinary ICH in constantly adapting to changes in the social environment, providing a quintessential case for research on the digital expression and sustainable transmission of regional culinary ICH.

### 3.3 Cultural resource collection

**Figure 2:**
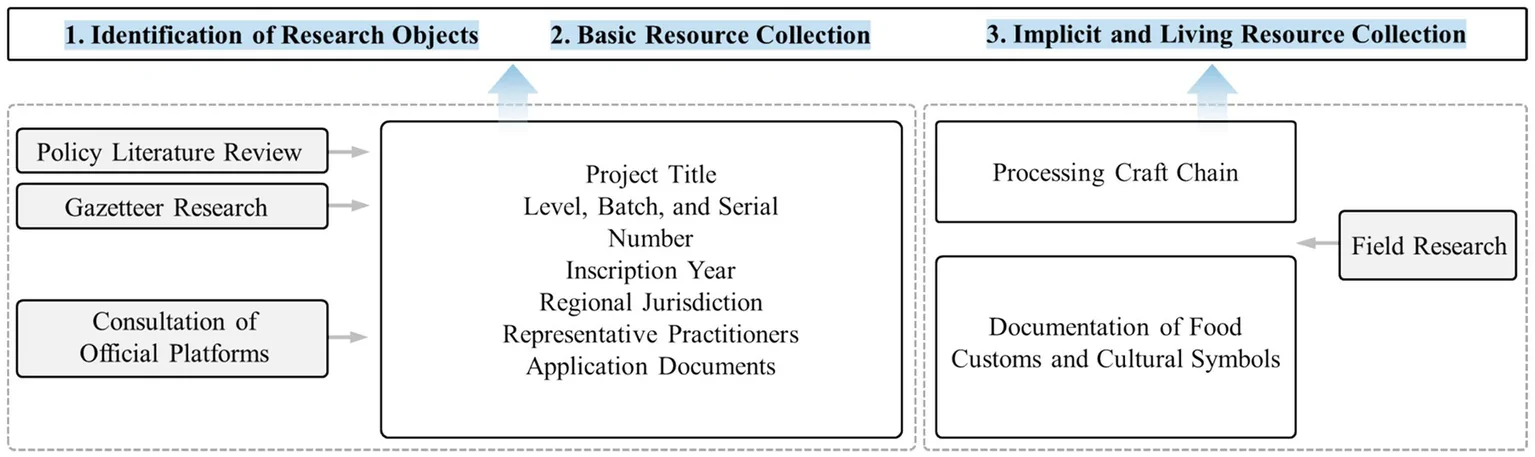
Collection path of culinary ICH resources in Hubei Province.

**Figure 3:**
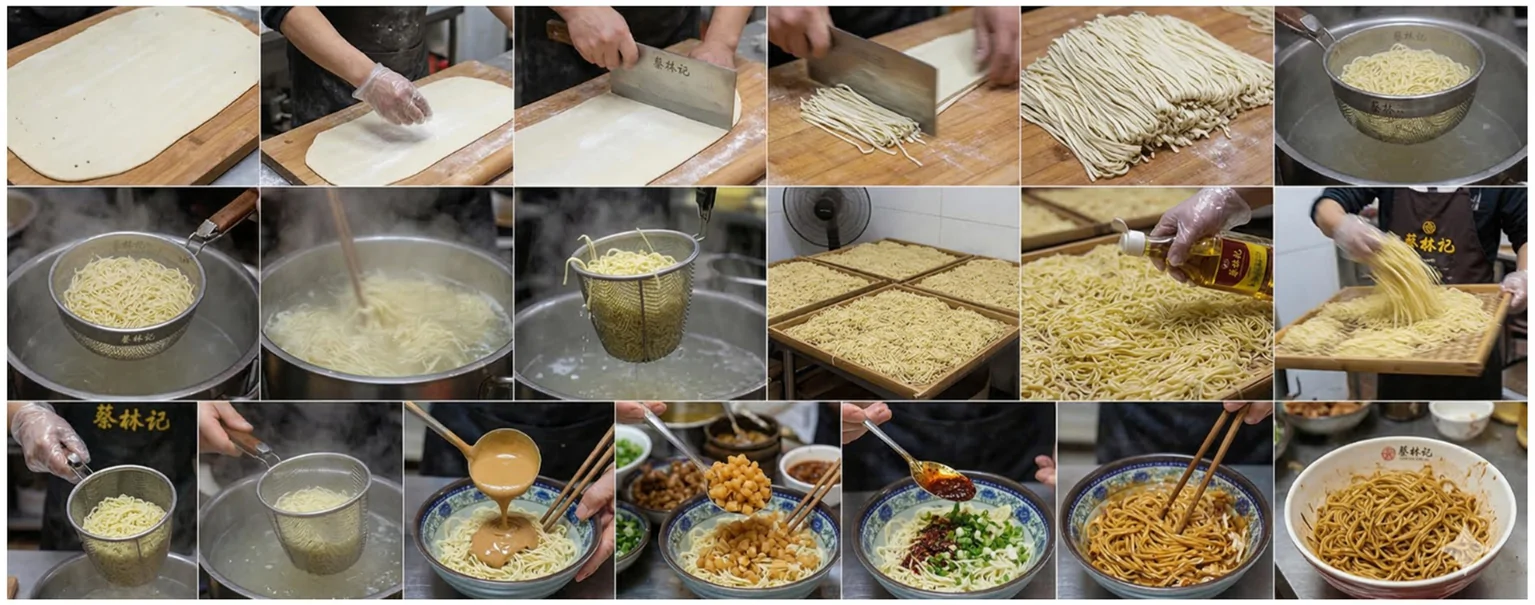
Collection records of key production processes.

In accordance with the design innovation strategies for the digital sustainable transmission of culinary ICH, cultural resource collection centered on Hubei Province’s culinary ICH was divided into three stages: identifying research subjects, collecting basic resources, and investigating implicit and living resources (as shown in Figure 2). Specifically, first, 50 research targets were identified based on the Representative Items of National Intangible Cultural Heritage of China. Second, basic in-formation on culinary ICH was obtained by consulting the official website of the Hubei Provincial Department of Culture and Tourism (44), the China Intangible Cultural Heritage Network (45), and public platforms of various municipal cultural centers, supplemented by cross-referencing policy and local chronicles such as the Hubei Provincial Chronicles: Trade (46) (as shown in Table 2). Subsequently, field visits were conducted to further acquire details difficult to present in official documentation, such as production chains and culinary symbols, particularly the implicit and living information of culinary ICH. Taking Cai Lin Ji hot-dry noodles as an example, the author visited multiple branches of Cai Lin Ji at the Yellow Crane Tower and Jiqing Street. In these production scenarios, operational judgments of key processes and the forms of implements were collected (as shown in Figure 3), and the local “Guo Zao” breakfast customs in Wuhan were recorded. Finally, the acquired information was summarized and organized. Through deduplication and merging, nearly 600 structured samples were obtained and stored in Excel, forming the basic database for Hubei Province’s culinary ICH.

**Table 2:** Sources of basic resources.

Category	Document name
Policy literature	List of China’s National Intangible Cultural Heritage Representative Projects (Batches 1–5) (45)
	List of Representative Intangible Cultural Heritage Projects of Hubei Province (Batches 1–7) (53)
	Law of the People’s Republic of China on the Protection of Intangible Cultural Heritage (2011) (54)
	Convention for the Safeguarding of the Intangible Cultural Heritage (2003) (1)
Local chronicles	Hubei Provincial Gazetteer: Trade (46)
	A Study of Jingchu Culinary Culture (55)
	Chronicles of Wuhan: Customs and Traditions: Dietary Habits (56)
Official platforms	Hubei Provincial Department of Culture and Tourism Official Website (57)
	Hubei Provincial Intangible Cultural Heritage Network (53)
	China Intangible Cultural Heritage Network (45)
	Wuhan Municipal Department of Culture and Tourism Official Website (58)

### 3.4 Resource classification

**Figure 4:**
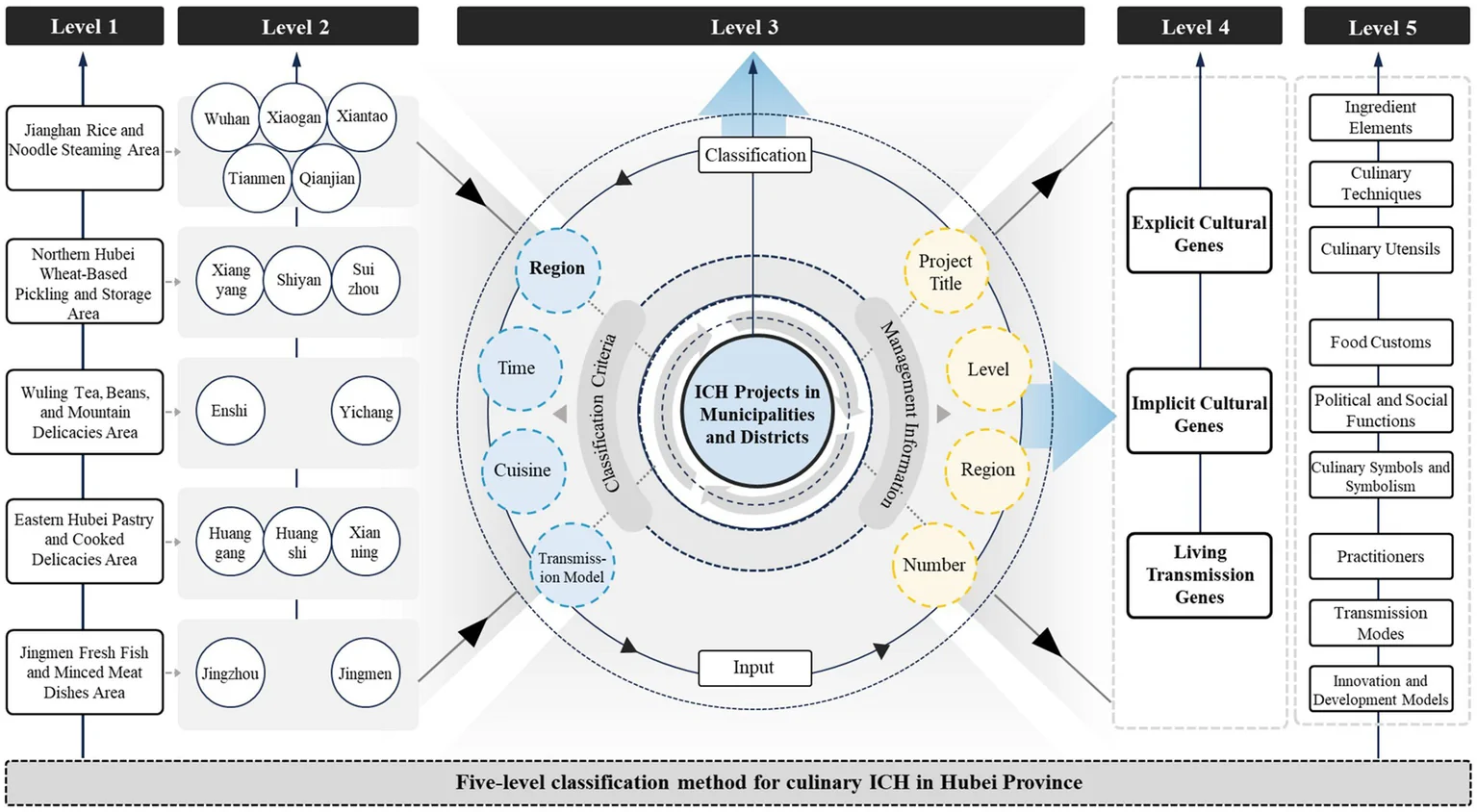
Five-level classification method for culinary ICH in Hubei Province.

A clear and rational data classification method is the cornerstone of database architectural design. While hierarchical classification helps address the diversity and complexity of cultural resources, traditional approaches often categorize cultural levels based on geography, administrative hierarchy, and project attributes (47), making it difficult to reveal the culinary ICH connotations beneath regional diversity. Culinary heritage should integrate multidimensional information—including culinary techniques, cultural connotations, historical backgrounds, and transmission processes—through structured knowledge organization, thereby achieving the systematic expression and digital presentation of culinary heritage. Therefore, building upon hierarchical classification and cultural gene theory, and integrating the specific regional distribution characteristics of Hubei culinary ICH, this research pro-poses the five-level classification method for culinary ICH, structured as “Level 1: Regional Zones—Level 2: Municipal Subdivisions—Level 3: ICH Projects—Level 4: Gene Categories—Level 5: Cultural Factors,” to systematically organize Hubei Province’s culinary ICH projects. Specifically, the method first divides Hubei’s culinary landscape into regional zones based on geographical characteristics: the Jianghan Plain core area, the Northern Hubei mountains and hills, the Wuling Mountains in southwestern Hubei, the Eastern Hubei hills, and the western Jianghan Plain. Second, administrative units at the municipal level with typical culinary styles within these regions are selected as the second-level classification. Specific ICH technique projects constitute the third level. The three major gene categories—explicit cultural genes, implicit cultural genes, and living transmission genes—form the intersecting fourth level. Finally, these genes are further refined into the fifth level, comprising cultural factors such as ingredients, culinary techniques, production processes, cultural connotations, and trans-mission modes (as shown in Figure 4). This classification method provides a scientifically sound methodological reference for the structured classification and storage of culinary ICH knowledge.

### 3.5 Metadata standard formulation

**Figure 5:**
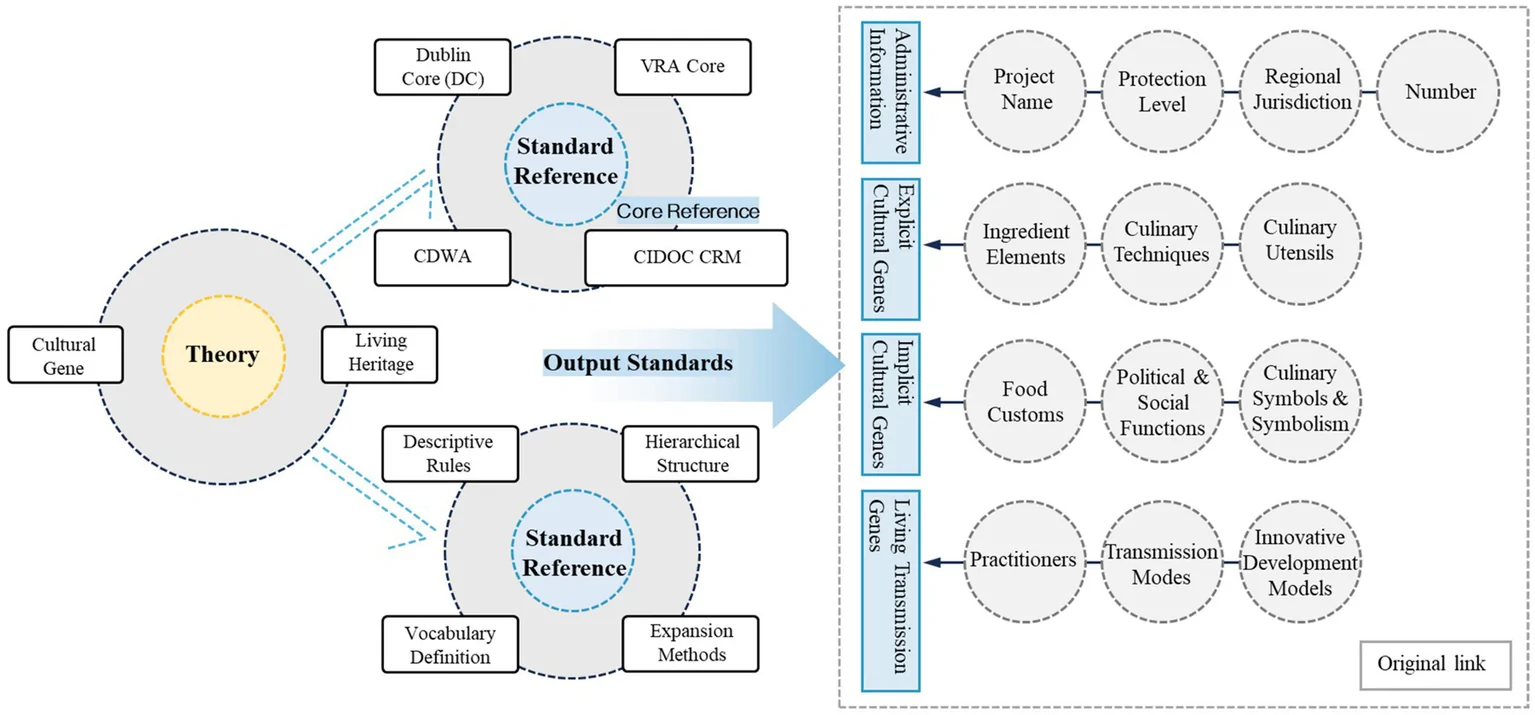
Metadata standard formulation for culinary ICH in Hubei Province.

To further refine the structured information organization of regional culinary ICH resources, corresponding metadata standards were formulated. Specifically, at the metadata level, international descriptive standards such as Dublin Core (48) were referenced. At the semantic level, the CIDOC-CRM (Conceptual Reference Model) (49) was introduced to guide the semantic relationship modeling among people, places, events, and cultural elements (49). Integrating the practical characteristics of culinary ICH, a domain-extended structure was constructed, encompassing explicit cultural genes, implicit cultural genes, and living transmission genes (as shown in Figure 5). This model emphasizes the semantic connections and relationship expressions of cultural heritage elements, enabling multi-dimensional systematic associations between entities. Consequently, it better aligns with the information architecture requirements of the five-level classification method for culinary ICH.

## 4 Case practice

### 4.1 Metadata encoding and annotation

Based on the established metadata standards and following a hierarchical encoding logic, Cai Lin Ji hot-dry noodles were classified and annotated into four categories: management information, explicit cultural genes, implicit cultural genes, and living transmission genes. These categories correspond, respectively, to the basic identity attributes, material expression, cultural connotation, and living transmission mechanisms of culinary ICH projects. Each type of metadata is encoded using the initials M, S, H, and A. The encoding follows the logic of “category letter + hierarchical index” to maintain the continuity of the five-level classification method for culinary ICH. The specific metadata annotation workflow is divided into the following steps: 1. Data Organization and Material Deduplication: The collected raw materials are unified into a structured format with source fields. After deduplication and merging, a dataset ready for annotation is formed; 2. Segmentation of Annotation Units: Based on the definition and scope of each metadata field, the organized data is segmented in Excel into project-level units, cultural-gene-level units, and their corresponding attribute fields, serving as the basic units for subsequent encoding and annotation; 3. Field Assignment and Code Generation: Unique identifiers are generated according to the “category letter + hierarchical index” encoding logic to ensure consistency in structure and hierarchy across different types of metadata; 4. Encoding Verification: The uniqueness of the codes is verified, and the consistency of the fields is checked.

Management information metadata is primarily used to identify the basic attributes and digital identity of ICH projects, with the aim of establishing a searchable and traceable standardized culinary ICH archive. This section covers contents such as project name, protection level, regional jurisdiction, and catalog number. These codes use the prefix “M,” serving both as the indexing prerequisite for the subsequent expansion of explicit, implicit, and living transmission genes and as the foundation for internal logical connection and systematic archiving within the database. As shown in Table 3, the recognition batch for Cai Lin Ji hot-dry noodle production techniques is annotated as M2, which is used to record the recognition batch and level of culinary ICH projects across different tiers. The geographical area where the project originated or primarily circulates is encoded as M3; the collection of this field assists academic researchers in conducting spatial-geographical analysis and regional cultural comparative studies of Hubei culinary ICH.

**Table 3:** Metadata annotation for management information of Cai Lin Ji hot-dry noodles.

Administrative information			
Label	Definition	Data Encoding	Example
Project name	The official name or common title of a culinary ICH project	M1	Cai Lin Ji hot-dry noodles
Protection level	Officially recognized level and approval batch	M2	Third batch of Provincial-level ICH
Regional jurisdiction	The geographic area where the project originated or mainly circulates, measured by City-District/County	M3	Wuhan City - Jianghan District
Number	The unique identification code recorded in the National ICH Directory	M4	149VII-13

Explicit cultural genes primarily focus on the material level of culinary ICH, serving as the direct manifestation of cultural genes in perceptible forms. These codes use the prefix “S,” covering subcategories such as culinary techniques, ingredient elements, and implements, which are further subdivided. For example, cultural factors under production techniques are annotated as S1-1 to S1-7 according to the workflow sequence, where S1-x represents the x operational step (x = 1–7). For each step, three attribute fields are simultaneously annotated: S1-x-1 (step description), S1-x-2 (operational details), and S1-x-3 (quality control judgment), to record the details of the production process in depth. Among explicit cultural genes, production techniques are the most intuitive cultural genes of culinary ICH; the precise annotation of their chains can reconstruct the daily operational logic and cultural experience of culinary inheritors. The production process of Hubei Cai Lin Ji hot-dry noodles unfolds sequentially from kneading, rolling, and cutting to boiling, “dan-mian”(Oil-tossing and Cooling), re-boiling, and seasoning. Due to the large volume of information in the production chain, each step contains operational details and quality control judgments, requiring corresponding sub-category codes and descriptions during the annotation process (see Table 4). Taking the core technique “dan-mian” as an example, the corresponding operational detail for this stage is “mix evenly from bottom to top,” and its quality control judgment is “strands remain distinct, loose, and non-sticky,” which are recorded as text values in fields S1-5-2 and S1-5-3, respectively.

**Table 4:** Metadata annotation for explicit cultural genes of Cai Lin Ji hot-dry noodles.

Explicit cultural genes——culinary techniques (S1)							
Step name	Kneading the dough	Rolling the dough	Cutting the noodles	Boiling	Oil-tossing and cooling	Re-boiling	Seasoning
Data encoding	S1-1	S1-2	S1-3	S1-4	S1-5	S1-6	S1-7
Description S1-x-1	Mix alkaline water with flour and knead	Roll the dough into a large sheet of uniform thickness	Cut the dough sheet into noodles of uniform thickness	Boil the noodles in boiling water	Spread noodles to cool quickly and drizzle with oil	Quickly blanch in boiling water to heat, then scoop out	Add sesame paste, soy sauce, and various other seasonings
Operational detail S1-x-2	Add alkaline water in batches; repeatedly knead and press	Fold into three layers and roll	Control thickness at approximately 1.75 mm	Boil for approx. 80–90 s; about 80–90% cooked	Toss and mix evenly from bottom to top	15–20 seconds	Pour the sauce first, then add noodles, and finally sprinkle with side ingredients
Quality control S1-x-3	Smooth dough surface, clean bowl, and clean hands	Uniform thickness without any breakage	No sticking between strands (clean cuts)	Noodles do not stick	Each strand is distinct; loose and non-sticky	Flexible, chewy, and elastic when scooped up	Sauce coats evenly; glossy with oil and not watery or runny

Explicit cultural genes——ingredient elements/culinary utensils						
Label	Definition	Data Encoding	example			
Ingredient elements	The primary raw materials required for the production of the Culinary ICH project	S2	Main ingredients S2-1	Alkaline noodles	Seasonings S2-2	Sesame paste, soy sauce, chili oil, aromatic vinegar
			Side dishes S2-3	Dried radish Pickled green beans		
Culinary utensils	Important tools during the production process	S3	S3-1	Deep pot	S3-2	Long-handled skimmer
			S3-3	Noodle tongs	S3-4	Long chopsticks

Implicit cultural genes primarily manifest the connection modes between cuisine and social life, cultural symbols, and collective memory. As shown in Table 5, within the dimension of food customs, hot-dry noodle is embedded in Wuhan’s unique “Guo Zao” culture, reflecting the tight correlation between citizens’ daily dietary behaviors and the regional rhythm of life. In the dimension of political and social functions, the “Wuhan City Business Card” is encoded as H2-1 under its subcategory, appearing extensively in government promotion and urban branding; consequently, hot-dry noodle is frequently endowed with the role of a cohesive force and spiritual symbol during major social events (43). Within the dimension of culinary symbols and symbolism of hot-dry noodles, the subcategory “dock culture and pragmatism” embodies the group temperament of local cuisine, while the culinary cultural symbol of “Wuhan people’s identity” highlights its deep-seated binding relationship with local identity.

**Table 5:** Metadata annotation for implicit cultural genes of Cai Lin Ji hot-dry noodles.

Implicit cultural genes			
Label	Definition	Data encoding	Example
Food customs	Festivals, rituals, and daily habits related to the cuisine	H1	“Guo Zao” (Wuhan Breakfast Custom)
Political and social functions	The role and value of food in political activities, social organizations, and group identity	H2-1	Wuhan City Business Card
		H2-2	“Wuhan Spirit” and Urban Resilience
Culinary symbols and symbolism	The connotations carried by food within the system of cultural symbols	H3-1	Dock Culture and Pragmatism
		H3-2	Identity of Wuhan People

Living transmission genes emphasize the dynamism and continuity of culinary ICH transmission. As shown in Table 6, these codes use the prefix “A” and cover three dimensions: inheritors, transmission modes, and innovation development models. The inheritor field reflects the genealogical chain of Cai Lin Ji hot-dry noodles as it evolved from family-based to enterprise-based transmission, providing a reference for analyzing the intergenerational succession of culinary ICH. The innovation development model reflects the regeneration paths of this ICH project within the context of modern consumption.

**Table 6:** Metadata annotation for living transmission genes of Cai Lin Ji hot-dry noodles.

**Living transmission genes**			
Label	Definition	Data encoding	Example
Inheritor	Representative practitioners of the project	A1-1	Cai Mingwei (Founder)
		A1-2	Cai Dasen (Eldest Son)
		A1-3	Cai Hanwen (Eldest Grandson)
		A1-4	Wang Yongzhong (Provincial-level representative practitioner)
Transmission modes	The methods of passing down the craft, such as family-based, apprenticeship, corporatized, or school education, etc.	A2-1	Family system
		A2-2	Apprenticeship system
		A2-3	Corporatized transmission
Innovative development models	Heritage and regeneration methods integrated with modern catering, tourism, digitalization, and other innovative paths.	A3-1	Integrated online-to-offline (O2O) Omni-channel Model
		A3-2	Cross-over co-branding consumption scenarios
		A3-3	Internationalization of craft via overseas store expansion

### 4.2 Requirement analysis for cultural gene visualization maps

To explore visualization formats adapted to the design innovation strategies for the digital sustainable transmission of culinary ICH, this section conducts research on the visualization methods and interactive functions of existing cultural resource databases and performs user requirement analysis. It explores the presentation format of culinary cultural gene visualization maps centered on knowledge graphs.

#### 4.2.1 Requirement analysis of Hubei culinary ICH gene visualization maps

To gain an in-depth understanding of the requirements of potential users for culinary ICH gene visualization maps, Accordingly, five visualization formats were identified: grid lists, regional maps, relationship diagrams, timelines, and multi-dimensional data charts. Furthermore, five functional dimensions for visualization maps were defined: knowledge interconnection, label annotation, language switching, comparative analysis, and user interaction. A questionnaire on visualization map formats and functional requirements was developed using a 5-point Likert scale. Quantitative analysis was performed on the general requirements of users when interacting with cultural resource databases to determine the final design presentation format. The questionnaire respondents consisted of individuals who use cultural resource visualization maps as potential research or design support tools. Specifically, this included 35 faculty members and postgraduate students with backgrounds in anthropology, sociology, or design from universities in Hubei Province, as well as 45 visual designers from the catering industry and food brands. The survey was distributed through a combination of offline and online methods; a total of 80 questionnaires were distributed, with 76 valid responses recovered.

Data analysis was conducted using SPSS, and the Cronbach’ *α* for the overall questionnaire was 0.878, indicating high internal consistency and favorable reliability for the scale. Through further data analysis, the functional requirements and visualization preferences of target users for culinary ICH visualization maps were identified (see Table 7). Regarding the visualization requirements for the culinary ICH database, respondents assigned relatively high importance ratings to “map-based cultural genes,” “timeline-based evolution visualization,” and “regional heatmaps,” reflecting a high level of emphasis on gene correlation, spatiality, and temporal visualization. In the dimension of visualization map functional preferences, “support for multi-project comparative analysis” and “knowledge interconnection of cultural genes” received the highest scores.

**Table 7:** Data analysis results of user requirements for Hubei culinary ICH gene visualization maps.

**Dimension**	**Name**	**Mean**	**Standard deviation**	**Rank**
Visualization dimension	Map-based cultural genes	3.908	0.836	2
	Multi-dimensional data visualization display	3.513	1.039	4
	Displays the complete production chain of ICH project techniques	3.500	0.973	5
	Supports timeline-based visualization of the evolution of ICH projects	3.882	0.979	3
	Supports geographic heatmaps to show the regional distribution of ICH projects	3.947	0.992	1
	Grid list	3.487	1.013	6
	I believe excessive visualization displays may reduce usage efficiency	3.461	1.064	7
Functional dimension	Knowledge interconnection of cultural factors	3.763	0.907	1
	Data tag annotation	3.526	0.973	4
	Establish a “Community Interaction Zone” to enhance user engagement	3.408	1.061	7
	Use “Map Visualization” to search for ICH projects	3.711	0.950	3
	Support multi-language switching for easier cross-cultural comparison	3.487	1.026	5
	Support “Multi-project Comparative Analysis”	3.737	0.885	2
	Excessive functional partitions on the page will interfere with research efficiency	3.447	1.088	6

In summary, user requirements for visualization presentation necessitate the selection of regional, temporal, and relational analysis methods, while functional requirements must focus on comparative analysis and knowledge interconnection. Therefore, knowledge graphs, Sankey diagrams, and spatiotemporal evolution maps were selected as the specific design presentation formats for the culinary ICH visualization maps. Among these, knowledge graphs are used to display the knowledge interconnection structure between cultural genes; Sankey diagrams focus on presenting the comparative relationships and factor flow relationships between multiple projects; and spatiotemporal evolution maps emphasize the evolutionary process of culinary culture across geographical and temporal dimensions. These three visualization formats complement each other, collectively supporting the systematic expression of explicit cultural genes, implicit cultural genes, and living transmission genes.

### 4.3 Cultural gene visualization maps

#### 4.3.1 Design and presentation of Hubei culinary ICH gene visualization maps

**Figure 6:**
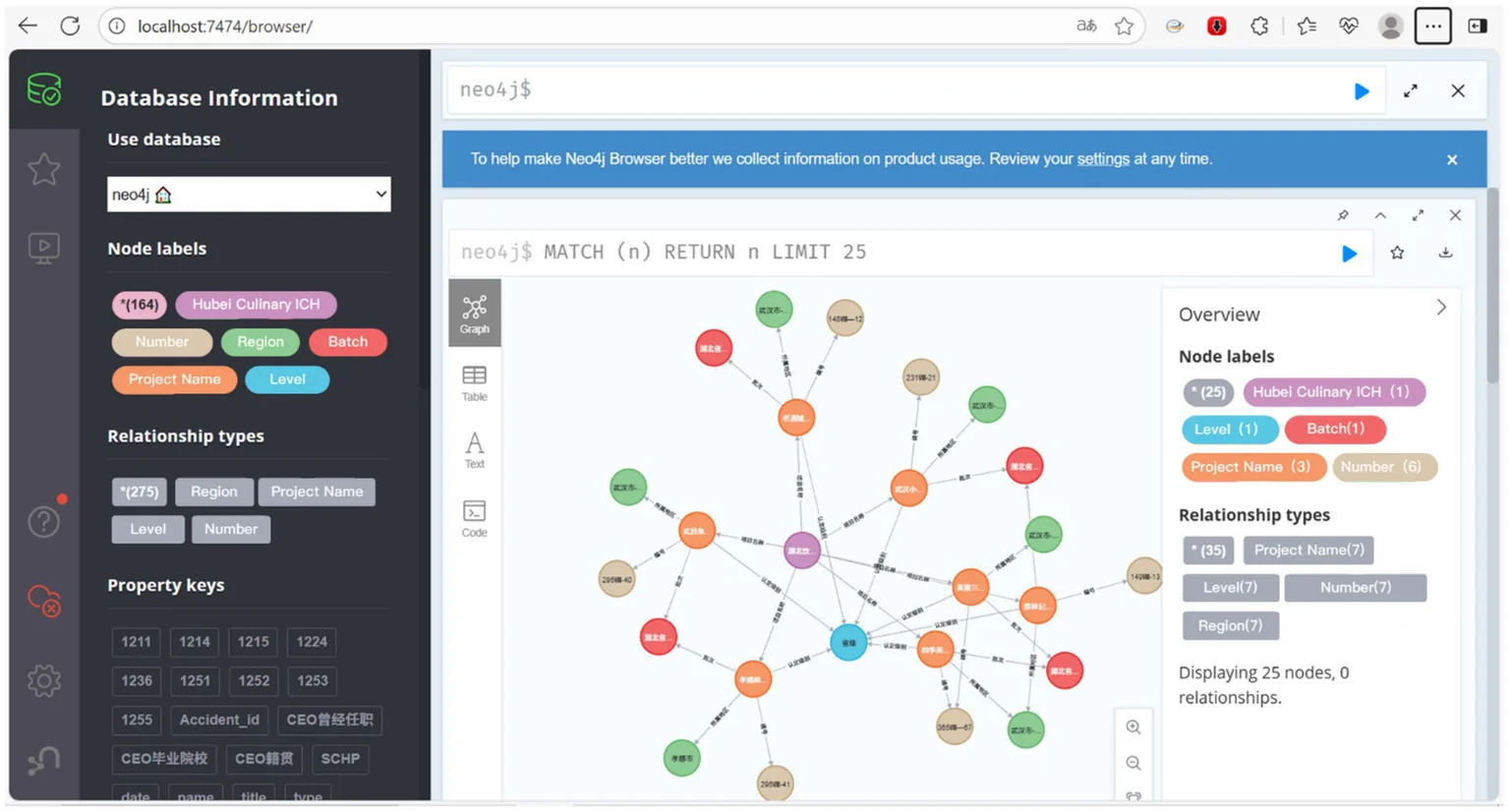
Construction of knowledge graphs for Hubei culinary ICH genes.

Further design practices were conducted by combining the previously determined visualization presentation formats. The map interface uses a front-end visualization framework as the presentation carrier. For the back-end of the knowledge graphs, a Neo4j graph database is utilized to implement the storage of semantic association data (see Figure 6).

**Figure 7:**
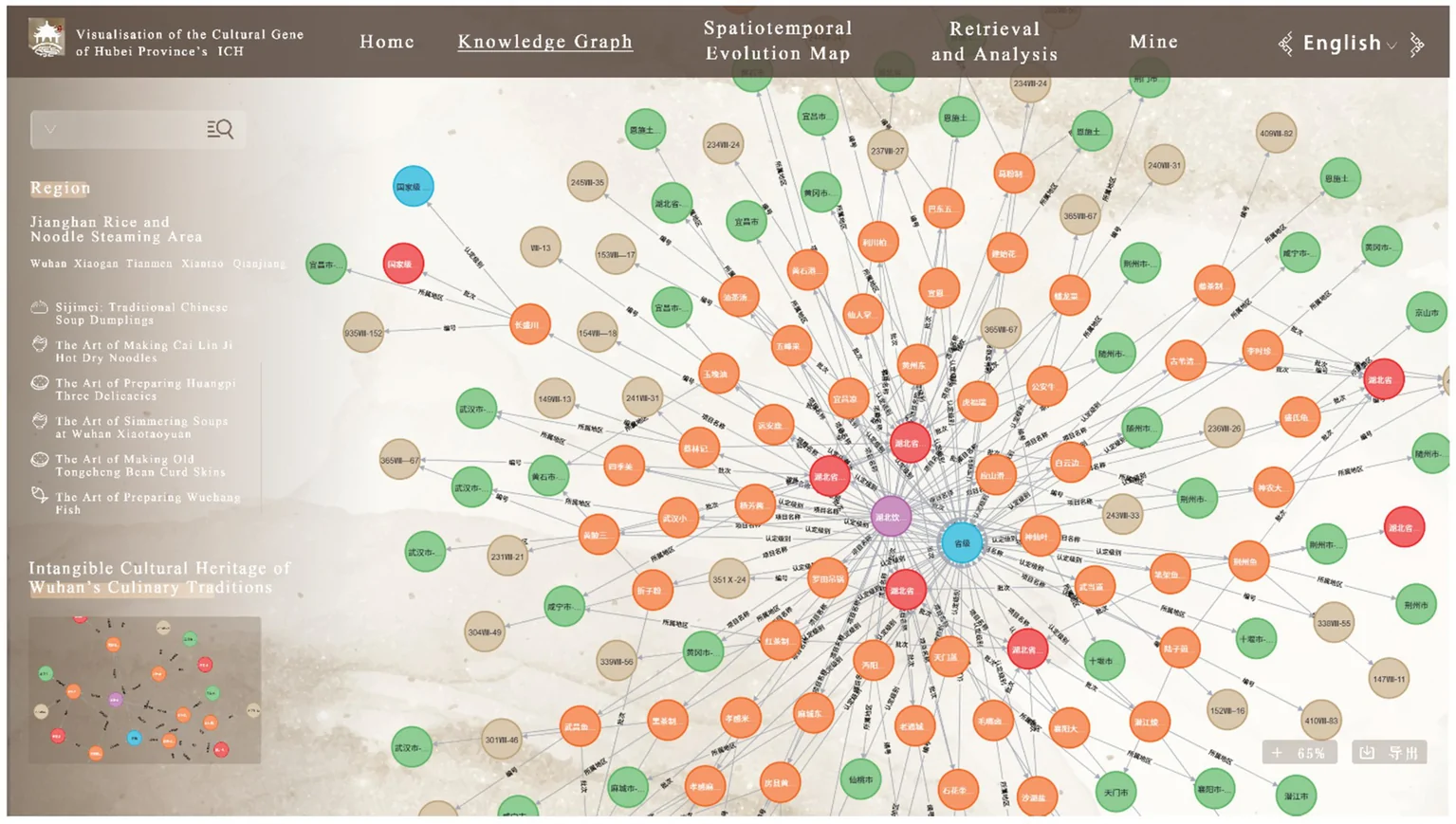
Knowledge graph of Hubei culinary ICH.

As the core research tool, the knowledge graphs for Hubei culinary ICH follow a logical structure based on the five-level classification method for culinary ICH. It integrates explicit cultural genes, implicit cultural genes, and living transmission genes—including regional distribution, technique chains, implement applications, and cultural symbols—into a unified knowledge network (see Figure 7). Researchers can flexibly switch observation perspectives through a dropdown filter function; for instance, filtering by a specific regional partition can intuitively display the semantic relationships of culinary ICH projects within a single area. This information association method based on knowledge graphs enhances the readability of the structure and logic of culinary ICH cultural resources, addressing the limitations of traditional text-based knowledge bases in the comparative analysis and interpretation of culinary ICH.

**Figure 8:**
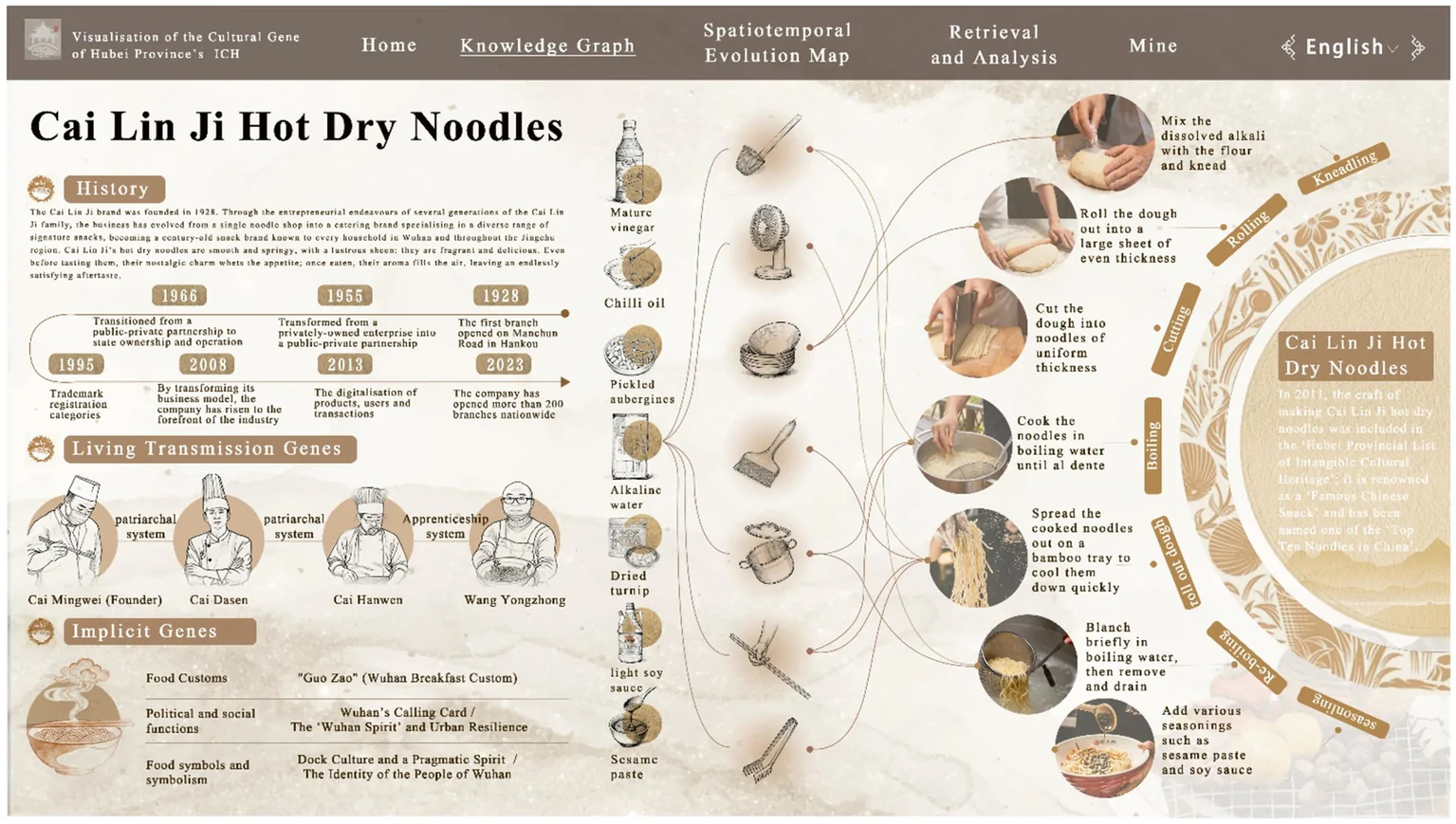
Knowledge graph of Cai Lin Ji hot-dry noodle cultural genes.

Taking the “Cai Lin Ji hot-dry noodle” as an example, the information visualization maps are based on the previously collected explicit cultural genes, implicit cultural genes, and living transmission genes. They demonstrate the information visualization effects of the culinary ICH project across these three dimensions, effectively assisting researchers in understanding the cultural connotation of culinary ICH in a simple and intuitive manner (see Figure 8).

**Figure 9:**
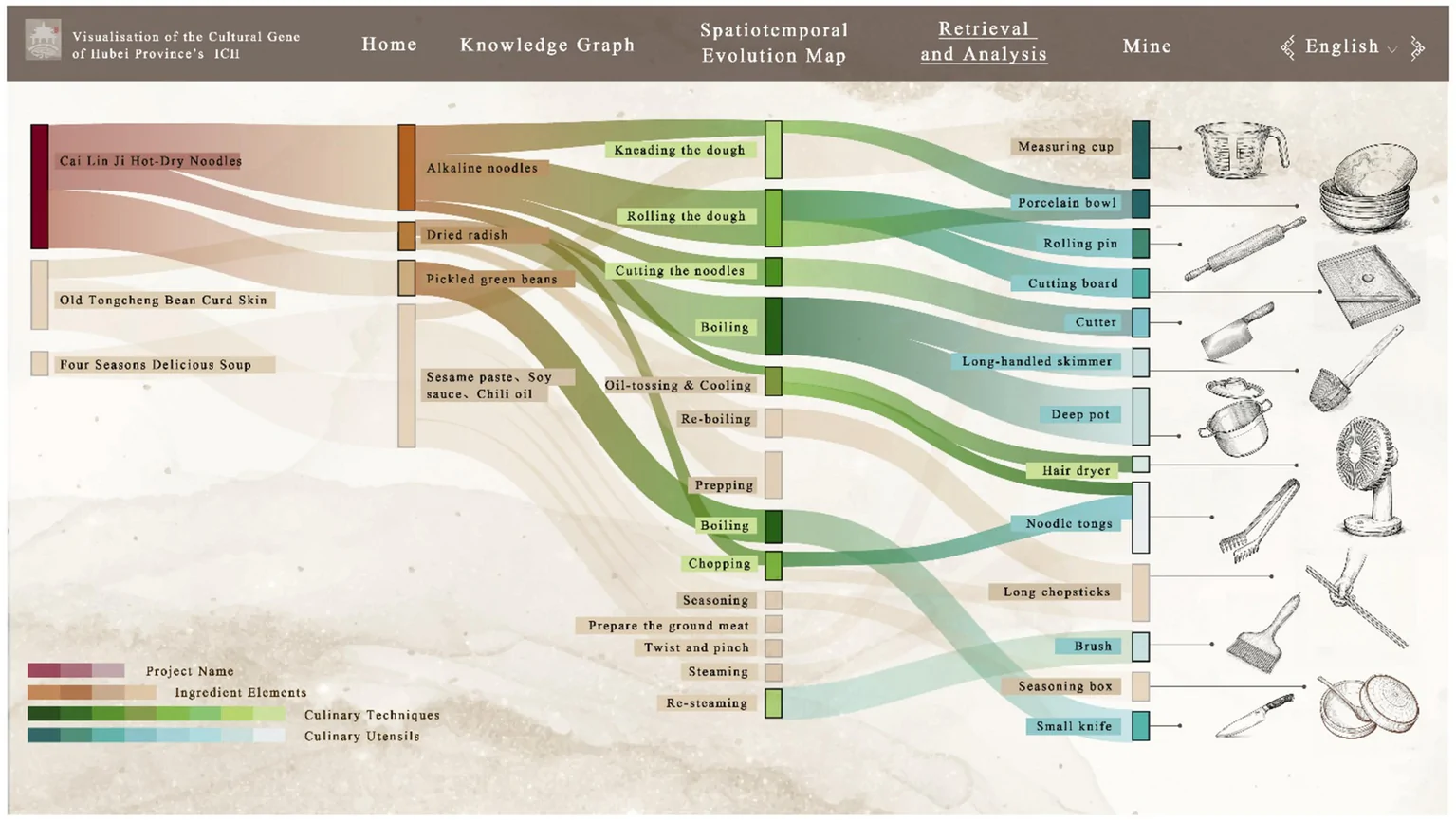
Sankey diagram.

Complementing the knowledge association network of knowledge graphs, Sankey diagrams and spatiotemporal evolution maps further expand the multi-dimensional analytical paths for culinary ICH research. The design logic of the Sankey diagrams page is based on a chain structure of “project name—ingredient elements—culinary techniques—utensils.” It presents the dependencies between the cultural genes of multiple culinary ICH projects through changes in flow, enabling a side-by-side comparison of the technological processes of various projects within a single visualization space. As shown in Figure 9, researchers can observe not only the production chain flow and cultural gene correlation of a single culinary ICH project but also clearly identify the differences in production methods and utensils usage among similar types of projects, as well as the commonalities in raw material selection. This multi-project production chain visualization provides intuitive evidence for horizontal comparison and reveals the characteristics of culinary ICH techniques in terms of procedural logic and implement usage, offering evidentiary support for researchers to conduct detailed project analysis and comparison.

**Figure 10:**
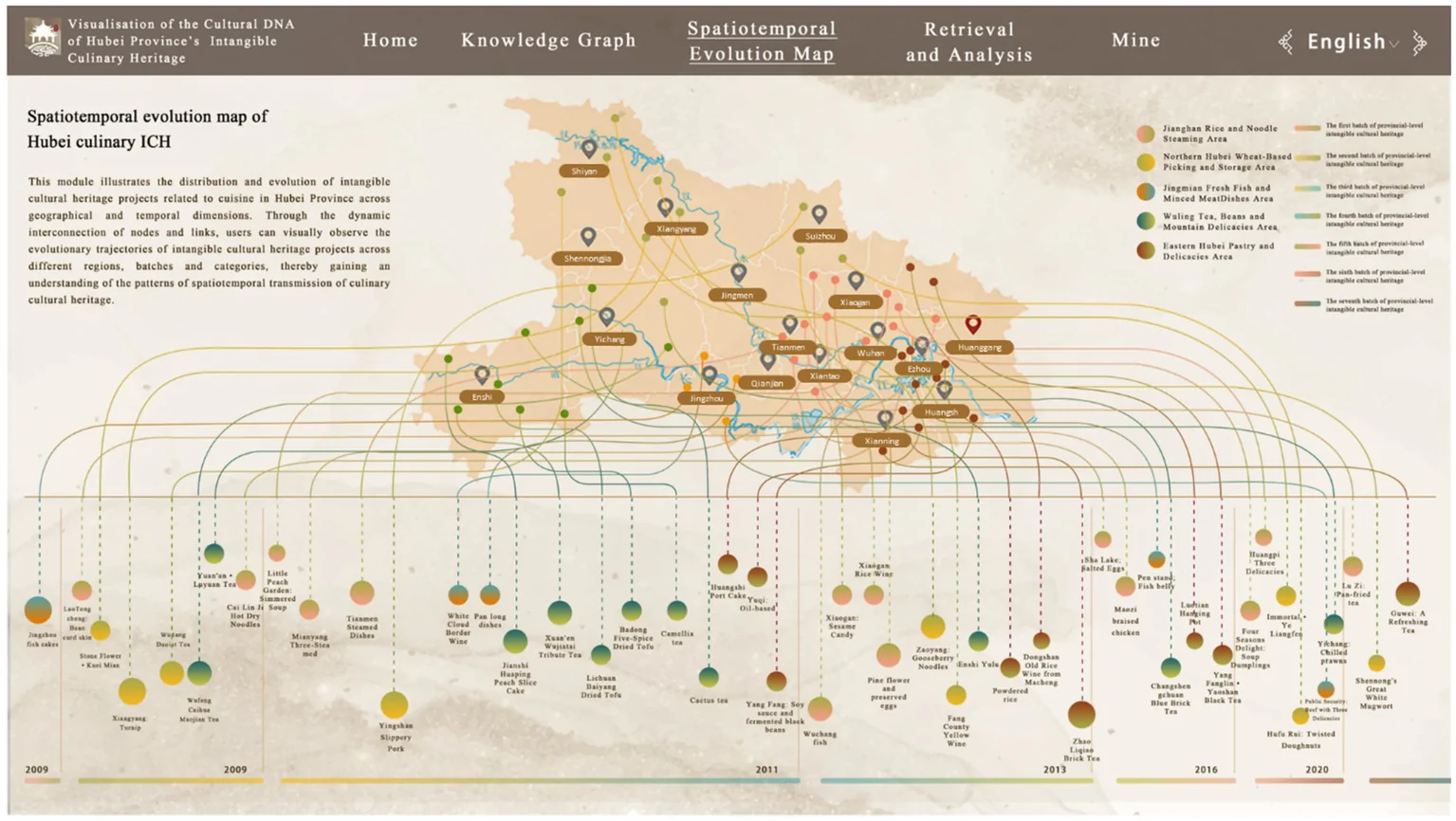
Spatiotemporal evolution map.

Spatiotemporal evolution maps center on the linkage between the map of Hubei Province and the declaration timelines and event flows of culinary ICH projects. Through the unified encoding of three elements—region, project batch, and time—these maps demonstrate the inclusion status and development trajectories of culinary ICH projects across different regions of Hubei Province during various historical stages (see Figure 10). Researchers can utilize spatiotemporal evolution maps from a macro perspective to grasp the spatial aggregation and diffusion levels of the province’s culinary ICH at different historical periods. Simultaneously, by selecting specific batches to trace relevant projects and tracking their cultural genes within the knowledge graphs, a dual investigation of the historical context and transmission methods of culinary ICH is achieved. The linkage mechanism among multiple visualization maps responds to the requirements identified in the earlier questionnaire research, such as “multi-project comparative analysis,” “support for regional heatmaps,” and “timeline-based evolution visualization,” assisting researchers in a complete and comprehensive understanding of the basic information, cultural connotation, and transmission mechanisms of culinary ICH.

## 5 Empirical verification of the design scheme

### 5.1 Experimental design

To verify the effectiveness of the design innovation strategies for the digital sustainable transmission of culinary ICH proposed in this study, this paper adopts an empirical research method. A comparative experiment was conducted between the visualization maps constructed based on these strategies and conventional information presentation methods. The aim was to investigate the impact of different information organization methods on users’ task completion efficiency and completion degree.

**Figure 11:**
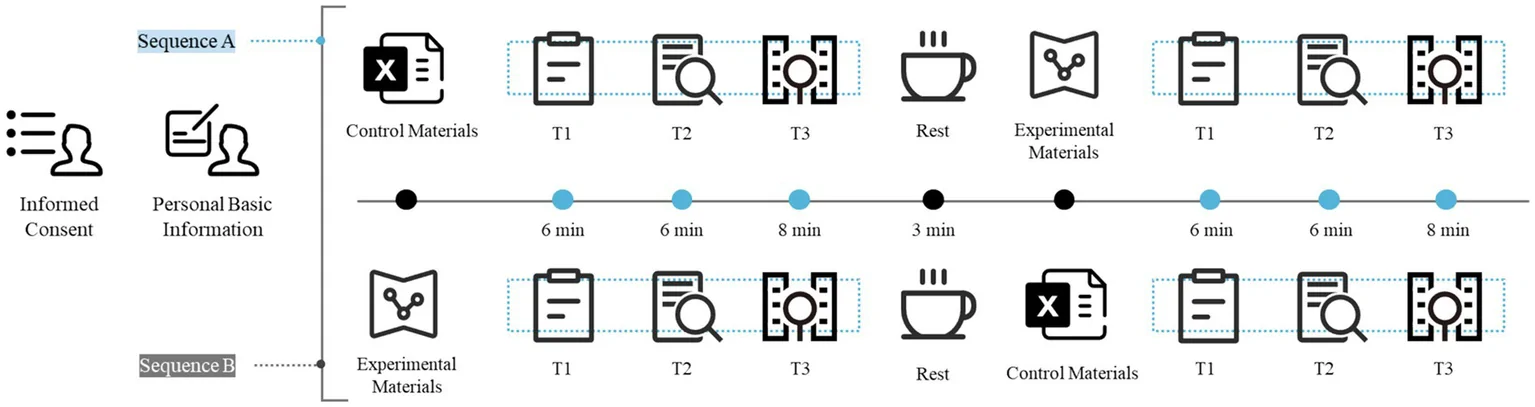
Experimental flow chart.

This study adopts a within-subjects design, meaning each participant completes the set experimental tasks using two different information carriers (50). A within-subjects design can effectively control for individual differences among participants and their impact on experimental outcomes, allowing the results to more directly reflect the experimental objectives (51). To avoid order effects, a counterbalancing method was applied to the experimental sequence (50). As shown in Figure 11, the experimental procedures for both groups remain consistent; participants were randomly assigned to two groups in a unified experimental environment. One group first completed the experimental tasks using the control materials, while the other group first completed the same tasks using the experimental materials. During the experiment, task completion time and performance results were recorded.

A total of 32 participants with academic or research backgrounds in design, culinary culture, ICH protection, or related interdisciplinary fields were invited for this study. Among the participants, there were 14 males and 18 females, with an average age of 24.6 years. The participants possessed fundamental cognitive abilities regarding themes such as cultural heritage protection, culinary culture, and ICH digitalization, enabling them to understand the experimental materials and complete the corresponding tasks.

### 5.2 Experimental materials and settings

**Figure 12:**
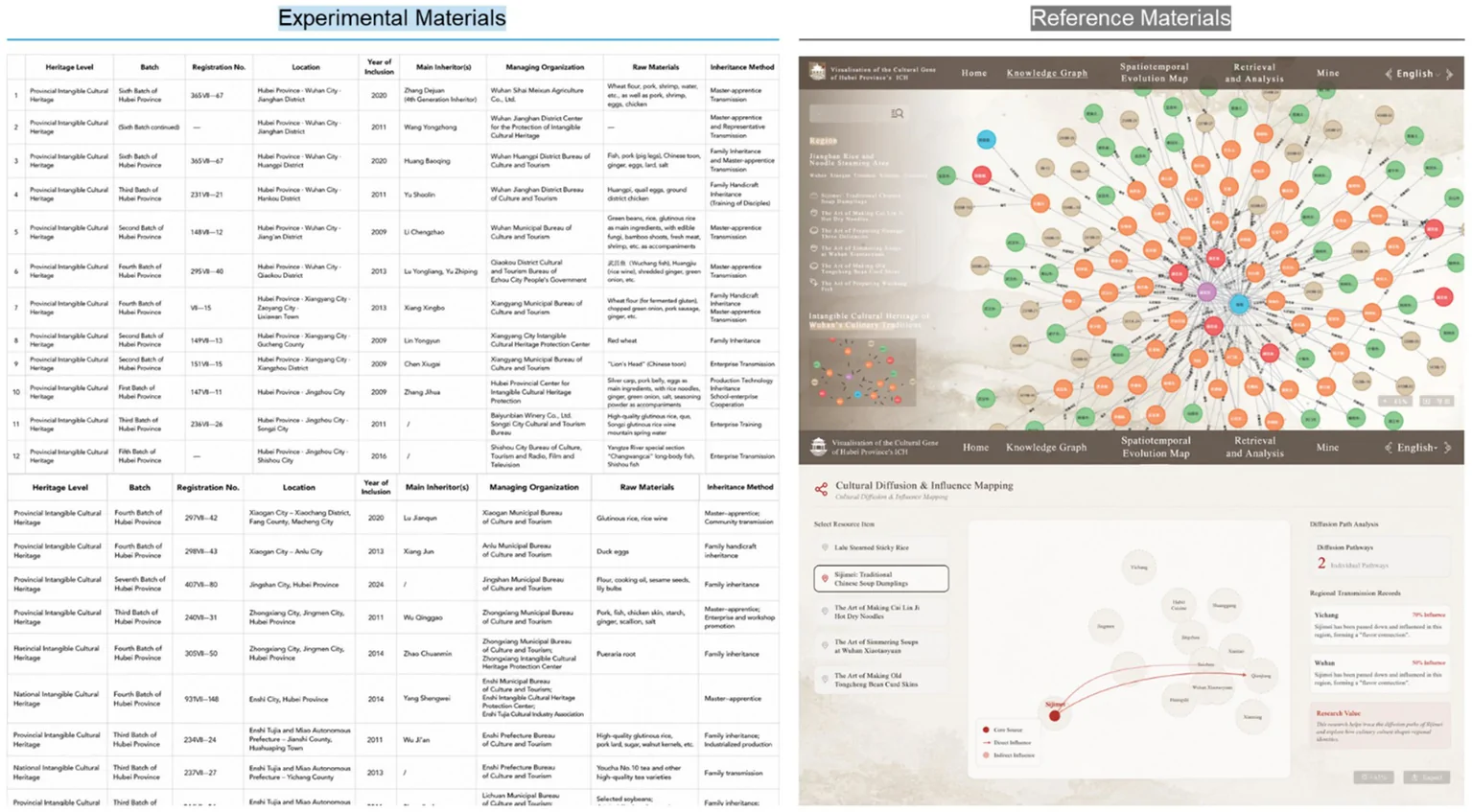
Comparison map of experimental materials.

This study set up a control experiment to compare the efficacy of participants using two types of information carriers to complete tasks. The two sets of experimental materials remained consistent in terms of cultural content sources and data scope, differing only in their information organizational structures and presentation formats (As shown in Figure 12).

The control materials primarily consist of Excel documents of Hubei culinary ICH resources compiled during the early stages of research and the China ICH Website. These materials present cultural information in the form of text lists, covering content such as project names, regional distribution, category attribution, production procedure descriptions, ingredient elements, and implements. This represents the most common information organization and presentation method currently found in culinary ICH exhibition platforms.

The experimental materials consist of the Hubei culinary ICH gene visualization maps constructed based on the design innovation strategies proposed in this study. Utilizing the five-level classification method for culinary ICH, a series of culinary cultural genes are presented through multi-dimensional formats, including knowledge graphs, procedure chain visualization, and spatiotemporal evolution maps.

### 5.3 Task design

Based on the transmission requirements of culinary ICH, this study set up three categories of experimental tasks to evaluate the practical role of different information organization methods in culinary ICH research. Participants were required to complete the tasks within a limited time (50 min). To avoid subjective judgment, the tasks and their evaluation objectives are shown in Table 8.

**Table 8:** Experimental task design and goals.

**Task**	**Description**	**Time**	**Evaluation objective**
T1	Organize the core production procedures of a designated culinary ICH project, clarify the sequential relationship of major operational steps	6 min	The impact of different information organization methods on the efficiency and accuracy of understanding production chain links
T2	Search for and summarize a list of culinary ICH projects under specific conditions (region/cuisine/time period)	6 min	The impact of different methods on the efficiency of retrieval and summarization of cultural resources
	Record the navigation path of results (Control Group uses Excel filters/keywords/row-column positions; Experimental Group uses Knowledge Graph view entries/relationship paths/node IDs)		Whether search results are reproducible and traceable
T3	Perform a comparative analysis of two designated culinary ICH projects of the same type; identify key differences in ingredient selection, culinary techniques, and culinary utensils	8 min	The role of different information organization methods in supporting comparative analysis and relationship judgment

### 5.4 Research hypotheses

Based on the experimental objectives of tasks T1, T2, and T3, as well as the anticipated advantages of the cultural gene visual organization approach under the proposed design strategies, the following research hypotheses are formulated:

*H1*: Compared with traditional information presentation modalities, the visual map of Hubei culinary cultural genes can significantly improve user efficiency in task completion.

*H2*: Compared with traditional information presentation modalities, the visual map of Hubei culinary cultural genes can significantly improve user effectiveness and accuracy in task completion.

### 5.5 Measurement indicators

This study established two categories of objective indicators: task completion time and accuracy rate. By calculating the completion time and score for each task, the differences between the two types of information carriers in supporting task execution efficiency and effectiveness were evaluated. Scoring was conducted based on pre-established unified scoring rules and standard answers to ensure the consistency of evaluation criteria.

#### 5.5.1 Task completion time

The time required for each participant to complete the three tasks (T1, T2, and T3) under the support of different information carriers was recorded. A shorter completion time indicates higher efficiency of the information organization method in supporting information retrieval and localization.

#### 5.5.2 Task scores

A unified 1–5 point scoring scale was adopted, with scoring independently conducted by two researchers and confirmed through consultation. The scoring criteria are as follow (Table 9).

**Table 9:** Task scoring criteria.

**Task**	**5 Points**	**4 Points**	**3 Points**	**2 Points**	**1 Point**
T1	Process sequence is completely correct	1 sequence deviation or process omission	2 sequence deviations or process omissions	3 sequence deviations or process omissions	4 or more sequence deviations or process omissions
T2	Completely correct	1 omission or error.	2 omissions or errors	3 omissions or errors	4 or more omissions or errors
T3	Identification is complete; expression is clear	1 identification error	2 identification errors	3 identification errors	4 or more identification errors

### 5.6 Empirical results

The study employed a one-way repeated measures analysis of variance (RM-ANOVA) to evaluate changes in user performance. Given that the experiment included a total of 32 participants (*N* = 32) and the independent variable comprised two levels, the corresponding effect degrees of freedom were determined as *k* - 1 = 1, and the error degrees of freedom within subjects were calculated as (*N* - 1) (*k* - 1) = 31. Consequently, the overall framework for inferential statistics was formally established with a degrees-of-freedom structure of *F* (1, 31).

The research results indicate that in terms of time indicators: in T1, the mean completion time for the experimental condition (*M* = 4.13, *SD* = 0.32) was significantly lower than that of the control condition [*M* = 5.25, *SD* = 0.27; *F*(1,31) = 228.4, *p* < 0.001, *η^2^* = 0.32]; in T2, the mean completion time for the experimental condition (*M* = 4.09, *SD* = 0.34) was significantly lower than that of the control condition [*M* = 5.02, *SD* = 0.18; *F*(1,31) = 167.3, *p* < 0.001, *η^2^* = 0.38]; In T3, the mean completion time for the experimental condition (*M* = 4.11, *SD* = 0.34) was significantly lower than that of the control condition [*M* = 7.00, *SD* = 0.27; *F*(1,31) = 1679.5, *p* < 0.001, *η^2^* = 0.43]. In summary, compared to the control group, the experimental group demonstrates a significant advantage in improving users’ task completion time efficiency.

Regarding the task completion capability scores, the research results indicate that: in T1, the mean task score for the experimental condition (*M* = 4.46, *SD* = 0.33) was significantly higher than that of the control condition [*M* = 3.63, *SD* = 0.38; *F*(1,31) = 87.2, *p* < 0.001, *η^2^* = 0.42); In T2, the mean task score for the experimental condition (*M* = 4.33, *SD* = 0.25) was significantly higher than that of the control condition [*M* = 3.58, *SD* = 0.36; *F*(1,31) = 88.9, *p* < 0.001, *η^2^* = 0.39]; In T3, the mean task score for the experimental condition (*M* = 4.00, *SD* = 0.37) was significantly higher than that of the control condition [*M* = 2.96, *SD* = 0.40; *F*(1,31) = 114.3,*p* < 0.001, *η^2^* = 0.46]. In summary, compared to the control group, the experimental group demonstrates a significant advantage in enhancing users’ task completion capabilities.

Furthermore, no order-related systematic bias was observed. In summary, the practical outcomes of the design innovation strategies for the digital sustainable transmission of culinary ICH demonstrate application feasibility in actual usage scenarios, verifying the effectiveness and operability of these strategies in real-world contexts.

## 6 Discussion

Aimed at the sustainable transmission and development goals of regional culinary ICH, this study proposes a systematic five-level classification method for culinary ICH based on cultural gene theory and living heritage theory. This method systematically classifies and integrates fragmented culinary ICH resources, constructing a five-level framework based on regional culinary cultural characteristics: “Level 1: Regional Zones—Level 2: Municipal Subdivisions—Level 3: ICH Projects—Level 4: Gene Categories—Level 5: Cultural Factors.” This classification method is similar to the digital resource classification for Xiangyu Nuo masks proposed by Xing Jianghao (52). However, it incorporates the “explicit-implicit dichotomy” into the classification hierarchy, effectively avoiding the phenomenon of focusing only on procedural chains while ignoring the cultural context during the resource collection process. Meanwhile, by including living transmission genes centered on inheritors, it accurately captures the dynamic characteristics of culinary ICH. This classification method achieves the precise extraction of explicit, implicit, and living transmission genes, helping to systematically and completely capture the deep cultural connotations and living characteristics of culinary ICH projects, thus effectively solving the difficulties in resource extraction and classification organization for culinary ICH.

The study proposes a design innovation strategy for the digital sustainable transmission of culinary ICH, encompassing cultural resource collection, resource classification, metadata standard formulation, metadata encoding and annotation, and cultural gene visualization map segments. At the level of culinary ICH sustainable development, this strategy transforms fragmented cultural resources into a structured knowledge system and multi-dimensional visualization maps with internal logic. Compared to single digitalization strategies that only study the construction and display of ICH knowledge graphs (10), this strategy integrates core stages such as systematic extraction and classification of culinary cultural resources, structured information organization, and visualization design translation. This ensures that culinary cultural genes can be precisely preserved in digital forms and applied innovatively, effectively preventing risks such as cultural context fragmentation and the dissipation of technique details during the modern digital transmission process; At the level of culinary culture sustainability, the systematic digital design strategy preserves and visually displays the production procedures, cultural semantic relationships, and transmission lineages of culinary ICH in a structured manner. Compared to previous simple compression storage methods based primarily on images and text, it can completely capture the authentic cultural forms of culinary ICH. Furthermore, the proposed strategy is adaptable to digital protection research for other regional culinary ICH, providing a foundation for experience exchange and methodological sharing between regions, as well as offering a reference for the long-term stable transmission and sustainable development of culinary ICH; At the level of social sustainability, through the multi-dimensional visualization maps of this strategy’s terminal, culinary culture researchers can conduct refined cultural resource retrieval and comparative studies across space–time and similar projects, improving research efficiency and effectively reducing the risks of redundant resource organization and cultural misinterpretation. Designers can use these maps to deeply understand the technique chains, cultural connotations, and living development processes of culinary ICH projects, thereby enhancing the efficiency of their design translation work. In the future, local cultural institutions, community members, and project inheritors can supplement and correct cultural resources under a unified data standard, strengthening multi-stakeholder participation and social synergy in the sustainable development of culinary ICH. At a deeper level, the visual map constructed through this strategy serves not only professionals but also charts a socio-cultural pathway for the general public to re-conceptualize the nutritional value and ecological balance of culinary ICH, achieved by transparently deconstructing regional dietary structures and culinary chains. This living awakening of regional dietary knowledge not only endows digital tools with a profounder technological-philosophical connotation but also provides an essential cognitive cornerstone for promoting sustainable food cultures among broader populations and guiding macroscopic behavioral shifts in dietary practices.

## 7 Conclusion

Against the background of the continuous advancement of China’s cultural digitalization strategy and ICH protection policies, this study takes Hubei culinary ICH as the research object to explore design innovation strategies for the digital sustainable transmission of culinary ICH. Through the extraction and classification of a large number of culinary ICH resources, the strategy constructs a five-level classification method for culinary ICH encompassing three categories of cultural genes: explicit, implicit, and living transmission genes. Combined with data encoding and labeling, it achieves systematic information organization of culinary ICH resources. Furthermore, relying on knowledge graph technology, it constructs multi-dimensional visualization maps, including Sankey diagrams and spatiotemporal evolution maps, providing a feasible strategic reference for the creative transformation and innovative development of culinary ICH in a digital context.

The study simultaneously reveals that current research on the digital sustainable transmission of culinary ICH still faces limitations, such as a restricted sample size, insufficient collection of living transmission resources, and the need to improve automated semantic extraction levels. Therefore, future research should expand in the following directions: First, expand the scale of resource collection by strengthening field investigations and community cooperation, incorporating more culinary ICH projects and inheritors’ oral history data into the resource organization workflow; Second, enhance the level of technical intelligence by introducing frontier technologies such as computer vision and knowledge reasoning algorithms. This will promote the automated and precise development of culinary ICH knowledge extraction and fusion, as well as multimodal recognition and semantic labeling, thereby improving the efficiency of resource classification and organization for culinary ICH.

Finally, it is necessary to cautiously define the boundaries of applicability for the findings of this study. The empirical experiment primarily validates that the culinary ICH visual map—as a practical outcome of the proposed strategy—improves short-term information utilization. Although this empirical evidence does not serve as a direct intervention test for the public’s real-world dietary behavior, from a long-term perspective, the structured resource management and multidimensional visual maps constructed through this strategy provide a long-term informational cornerstone and tool support for the digital preservation of culinary ICH. This can enduringly mitigate the risks of information redundancy and misalignment in cross-regional and cross-subject collaborative preservation, thereby supporting and serving the sustainable preservation process of macro-scale dietary culture over the long term.

## Data Availability

The original contributions presented in the study are included in the article/supplementary material, further inquiries can be directed to the corresponding author/s.
